# The Two Phenotypes of Renal Failure Associated with Chronic Urinary Retention

**DOI:** 10.3390/jcm15218680

**Published:** 2026-11-06

**Authors:** Ofer N. Gofrit, Vladimir Yutkin, Guy Hidas, Mohammad Zuaiter, Mordechai Duvdevani, Amitay Lorber

**Affiliations:** Department of Urology, Hadassah Hebrew University Hospital, P.O. Box 12000, Jerusalem 91120, Israel

**Keywords:** benign prostatic hyperplasia, chronic urinary retention, renal failure

## Abstract

**Background:** Renal failure (RF) is a severe complication of chronic urinary retention (CUR). In this study, we inquired whether RF associated with CUR is a single entity or comprises two different phenotypes: one with rapid onset and recovery after bladder catheterization and the other with gradual onset and limited reversibility. We also assessed whether these two phenotypes could be distinguished at presentation. **Methods:** This retrospective study included 161 men hospitalized from 2000 through 2025 with CUR and RF, defined as serum creatinine ≥2.0 mg/dL. **Results:** After catheterization, serum creatinine decreased to <2.0 mg/dL in 121 patients (75.2%; phenotype one) and remained ≥2.0 mg/dL in 40 (24.8%; phenotype two). The groups did not differ in age, bladder volume at catheterization or in prostate volumes. Among 49 patients with phenotype one who had a creatinine measurement during the preceding year, 48 had a normal value, supporting rapid development of RF. Mean admission hemoglobin was 12.7 g/dL (SD 1.9) in phenotype one and 9.8 g/dL (SD 1.9) in phenotype two (<0.0001). A hemoglobin cutoff of 11.7 g/dL predicted phenotype one with 73.6% sensitivity and 90.0% specificity; the area under the receiver operating characteristic curve was 0.888. **Conclusions:** We concluded that CUR-associated RF appears to comprise a rapidly reversible phenotype and a slowly developing, minimally reversible one. Admission hemoglobin may help distinguish these groups. We suggest that RF arises through two different pathophysiological mechanisms in the two types.

## 1. Introduction

Urinary retention can be described as complete or partial, acute or chronic, painful or silent, obstructive or non-obstructive, and associated with high or low bladder pressure. Chronic urinary retention (CUR) is of particular interest because it may be associated with renal failure (RF). To our knowledge, a standard definition of CUR has not been established [1]. Moreover, this uncertainty stems from the lack of consistent evidence that kidneys are in danger over a certain residual volume. Abrams et al. suggested defining CUR as post-void residual urine of >300 mL, which is the volume at which the bladder usually becomes palpable [2]. The American Urological Association guidelines went further and defined CUR as residual urine >300 mL measured twice over six months. This threshold was selected to reduce the risk of false-positive diagnoses resulting from overhydration or insufficient time for complete evacuation during testing [3]. However, other authorities have adopted higher thresholds, with the UK National Institute for Health and Clinical Excellence adopting a cutoff of >1000 mL [4].

The risk of RF in patients conservatively treated with CUR remains controversial. Abello et al. reported that under medical treatment, RF developed within 13 months in 14/28 men with CUR (defined by the authors as post-void residual >300 mL) [5]. Bates et al. followed 93 men with CUR (that they defined as post-void residual > 250 mL) treated medically and reported that RF developed in only three after a follow-up of 3 years [6].

In contrast to the dramatic and painful presentation of acute urinary retention, CUR typically develops insidiously and may remain clinically silent for several years. CUR initial presentation can include new-onset hypertension, peripheral edema, and RF. It is common for patients to present to a urologist for the first time while already experiencing RF.

In this study, we evaluated patients with CUR associated with RF. We inquired whether this is a single entity or does it encompasses two distinct phenotypes: one characterized by rapid onset and reversibility, and the other that develops insidiously and is often irreversible. We suggest that these two entities can be differentiated upon initial presentation and that they stem from different pathophysiological mechanisms.

## 2. Materials and Methods

### 2.1. Definition of Renal Failure

The standard definition of RF is a decrease in the glomerular filtration rate (GFR) to <60 mL/min/1.73 m^2^. GFR is best measured using inulin clearance; however, this is clinically impractical and serum creatinine-based predictions are usually used clinically. Different authors have used various cutoffs for serum creatinine levels to define RF. Mebust et al. used a serum creatinine cutoff >1.5 mg/dL, and Flanigan et al. used 3.0 mg/dL [7,8]. In the present study, eGFR and GFR category (G1–G5) were determined according to the KDIGO 2021 Clinical Practice Guideline for the Management of Blood Pressure in Chronic Kidney Disease [9]. For statistics, a cutoff of 2.0 mg/dL was chosen. This level signals a drop of 50% in GFR in most cases [10].

### 2.2. Definition of Urinary Retention

Urinary retention was defined as the need for urgent bladder catheterization. When bladder residual volume was found to be 300 mL or more, CUR was diagnosed according to Abrams et al. [2].

### 2.3. Study Population and Patient’s Management

This retrospective study involved a review of hospital records to identify patients diagnosed with both urinary retention (International Classification of Diseases, Ninth Revision, Clinical Modification [ICD-9-CM] code 788–20) and RF (ICD-9-CM code 586) between 1 January 2000 and 31 December 2025.

Male patients included in the study met all the following criteria: serum creatinine ≥ 2.0 mg/dL upon admission, urinary retention with post-void residual volume > 300 mL that requires urgent bladder catheterization, and hospital admission after bladder catheterization. Conversely, male patients who were excluded from the study met any one of the following criteria: recent exposure to nephrotoxins, bladder or prostate malignancy, history of surgery on the urinary system, urinary stones (anywhere in the urinary system), urethral stricture, and neurogenic bladder.

After bladder catheterization in the emergency department, all patients were hospitalized for urinary output monitoring and follow-up of electrolyte balance. Post-obstructive diuresis was defined as urine output >200 mL/hour for at least two consecutive hours. Cross-sectional imaging with ultrasonography or CT were done to evaluate the presence of hydronephrosis, prostate volume and other pathologies of the urinary system. Patients were discharged from hospitalization after subsiding post-obstructive diuresis. Recovery from RF was defined as a documented serum creatinine level < 2.0 mg/dL. Failure to improve was not considered until three months after bladder catheterization. All patients remained with an indwelling catheter until prostate surgery. Kidney function was evaluated after 3 months [11].

This study was approved by the Hadassah Medical Center Review Board (approval number: HMO-0279-26 from 11 October 2026) and adhered to the principles of the Declaration of Helsinki (2024). The board waived the requirement for informed consent as a retrospective study on anonymized data.

### 2.4. Statistical Analysis

Comparisons between patients with reversible (phenotype one) and irreversible (phenotype two) RFs were conducted using two-tailed t-tests for numerical data, Chi square test and Fisher’s exact tests for categorical data. The receiver operating characteristic (ROC) curve was used to evaluate the ability of the hemoglobin and creatinine levels upon admission to predict the CUR phenotype. The threshold for statistical significance was set at α = 0.05. This study complied with the STROBE checklist v4 for observational studies. All raw data are presented in Supplementary Materials. AI (ChatGPT-6.1) was used for preparation of figures and for minor language editing in accordance with MDPI’s guidelines.

## 3. Results

Between 1 January 2000 and 31 December 2025 7799 patients were hospitalized with a diagnosis of ICD-9-CM code 788-20 (urinary retention unspecified). Among these patients, 263 were also diagnosed with ICD-9-CM code 586 (RF, unspecified) and 161 met the inclusion and exclusion criteria. Patient’s characteristics are presented in Table 1, and their course and final fate in Figure 1. Upon admission, RF was severe with 90% of the patients presenting with KDIGO GFR categories G4 and G5, and an additional 10% with G3b.

During follow-up, 121 men (75.2%), serum creatinine level decreased to a nadir below 2.0 mg/dL. These patients were classified as “phenotype one.” In 40 men (24.8%), creatinine levels did not decrease below this threshold after a minimum follow-up of three months, and they were classified as “phenotype two”. Admission of serum creatinine and hemoglobin levels to the CUR phenotype are shown in Figure 2.

Table 2 summarizes the features of two phenotypes. They were similar in age, residual bladder volume, prostatic volume, and the presence of electrolyte or acid–base imbalances. Patients with type two had a higher frequency of hydronephrosis and lower tendency to develop post-obstructive diuresis. Upon admission to the hospital, the average serum creatinine level of patients with phenotype two was 6.3 mg/dL (SD 4.6) compared to 4.3 mg/dL (SD 2.6) in patients with type one (*p* = 0.01). However, a substantial overlap between the phenotypes was observed (Figure 2A). Average hemoglobin levels of patients with phenotype one was 12.7 g/dL (SD 1.9) compared with 9.8 g/dL (SD 1.9) in phenotype two (*p* < 0.0001). Only a small overlap was observed between groups (Figure 2B).

ROC analysis of the association between hemoglobin and creatinine levels at presentation and chance of renal recovery is shown in Figure 3. Hemoglobin provided an AUC of 0.888 (95% CI 0.820-0.944), while the AUC of creatinine was only 0.658 (95% CI 0.560-0.746). Hemoglobin levels higher than 11.7 g/dL predicted phenotype one with sensitivity of 73.6%, specificity of 90%, and Youden’s J of 0.636.

Among 49 men with phenotype one, serum creatinine measurements obtained <1 year before admission were available. In 48 of them (97.9%), the levels were <2.0 mg/dL, with an average of 1.2 mg/dL (SD 0.4). Upon admission, their average serum creatinine increased to 4.3 mg/dL (SD 2.5) and after catheterization, it decreased to 1.2 mg/dL (SD 0.4). In 26 of these men, normal serum creatinine levels were documented within one month before admission. Among men with phenotype two, serum creatinine measurements obtained within one year before admission were available for 19 patients, with an average of 2.0 mg/dL (SD = 1.0). Levels <2.0 mg/dL were documented in only 12 patients.

Post-obstructive diuresis occurred in 94.2% of the patients with phenotype one and 67.5% with phenotype two (*p* = 0.00005). Finally, 97 patients underwent transurethral prostatectomy (TURP), 27 open prostatectomy, 23 holmium laser enucleation of the prostate (HoLEP), and 15 remained with an indwelling catheter.

## 4. Discussion

This study explored the clinical presentation and course of RF, one of the most severe complications of urinary obstruction. RF upon admission was severe, with 90% of the patients presenting with KDIGO GFR categories G4 and G5, and 10% with G3b. After bladder catheterization, it was clear that CUR encompasses two distinct clinical phenotypes: phenotype one (75% of patients) is characterized by rapid development and reversibility, and phenotype two (25% of patients) by a slow, insidious course, and irreversibility. The two phenotypes were found to be similar in patient’s age at presentation, bladder volumes at catheterization, prostatic volumes, and presence of electrolyte or acid–base imbalances during management. Serum creatinine levels of patients with phenotype 2 were significantly higher, but with a substantial overlap with those with phenotype 1 (Figure 2A). Hemoglobin levels showed a significant difference between the phenotypes (12.7 ± 1.9 vs. 9.8 ± 1.9 g/dL, *p* < 0.0001), with only a small overlap (Figure 2B). Hemoglobin levels of 11.7 g/dL or higher predicted phenotype one with sensitivity of 73.6%, specificity of 90%, and Youden’s J of 0.636 (Figure 3A) and creatinine levels on admission provided no such prediction (Figure 3B). Normal creatinine levels were documented the year before admission in 48 of 49 men with phenotype one (in 26 men, normal creatinine levels were documented the month before admission), suggesting rapid development of RF.

Possible mechanisms underlying CUR-associated RF may explain why patients develop different phenotypes. Ten mechanisms of RF-associated CUR can be perceived:

1. A rise in bladder-filling pressure. While the normal filling pressure of the bladder is 0–6 cm H2O, during CUR, filling pressures may increase. When rising above 20–25 cm H2O, which is the pressure inside the ureteral peristaltic wave, the effectiveness of peristalsis decreases and the upper tract dilates, further compromising urine transport [12].
2. Obstruction of the ureterovesical junction. Detrusor muscle hypertrophy leads to functional and anatomical obstruction of the ureterovesical junction, compromising peristaltic effectiveness [13].
3. Decrease in net filtration pressure (NFP). GFR equals NFP minus net osmotic pressure. NFP is the difference between glomerular hydrostatic pressure and Bowman’s capsule pressure. When the pressure in the collecting system increases, it is transmitted to Bowman’s capsules, resulting in an increase in hydrostatic pressure, shutting down the GFR [14].
4. Ischemia of the thick ascending limb of Henle’s loop and juxtamedullary nephrons. Perfusion of the thick ascending limb of Henle’s loop and juxtamedullary nephrons is particularly vulnerable to increases in hydrostatic pressure within the collecting system. Ischemia in these structures impairs the medullary countercurrent mechanism and decreases the responsiveness of juxtamedullary nephrons to antidiuretic hormones. Both mechanisms lead to a decrease in the concentrating ability of the kidneys, resulting in nephrogenic diabetes insipidus [14].
5. Hypovolemia due to nephrogenic diabetes insipidus. Nephrogenic diabetes insipidus itself leads to chronic hypovolemia and kidney hypoperfusion [14].
6. Failure of the uretero-vesical junction valve mechanism. Chronically elevated bladder pressure alters the anatomy of the ureterovesical junction valve, leading sometimes to its failure [15]. Amar et al. showed that vesico-ureteral reflux is common in patients with obstructive prostate disease [16]. The collecting system, which normally operates at pressure levels of a few centimeters of H2O, is suddenly exposed to pressures several-fold higher. This pressure wave is transmitted through the collecting system to the distal and proximal tubules and to Bowman’s capsule, reducing the NFP and decreasing the GFR (as in the second mechanism above).
7. Renin–angiotensin activation. Exposure of the collecting system to high pressure activates the renin–angiotensin system, and the secretion of thromboxane A2. Both mechanisms induce renal vascular vasoconstriction, causing further kidney ischemia [14].
8. Tendency to recurrent infections and stone formation in patients with CUR.
9. Repeated events of acute urinary retention.
10. Secondary hypertension induces glomerulosclerosis leading to RF [14].

The biochemical and histological changes that occur during unilateral or bilateral ureteral obstruction have mainly been studied in animal models. Within hours of experimental obstruction, the kidneys experience mechanical stress, increased renal vascular resistance, reducing blood flow and GFR. Within days, inflammation sets in and profibrotic cytokines are released. After several weeks, myofibroblast accumulation is observed. Tubular atrophy and fibrosis follow. After several months, matrix deposition and tubulointerstitial fibrosis (TIF) set in [17]. Although animal models cannot fully simulate the clinical situation, renal biopsies from patients with irreversible obstruction-related RF demonstrated TIF [18]. At the molecular level, myofibroblasts are the principal cells responsible for excessive production of extracellular matrix. These cells originate both locally and in bone marrow. Local cells that transform into myofibroblasts include interstitial cells, epithelial cells that undergo epithelial-to-mesenchymal transition, macrophages that undergo macrophage-to-myofibroblast transition, endothelial cells that undergo endothelial-to-mesenchymal transition, and pericytes. Bone marrow–derived macrophages, especially profibrotic M2 macrophages, promote TIF development by secreting profibrotic factors, such as transforming growth factor-β [17]. The process ultimately leads to permanent disruption of kidney architecture and function, and recovery is not expected following relief of hydrostatic pressure.

Among the ten proposed mechanisms of CUR-associated RF, only one can potentially develop within hours to days and is reversible almost immediately upon pressure relief by bladder catheterization, as observed in phenotype one. This mechanism involves failure of the ureterovesical junction valve and development of vesicoureteral reflux (mechanism #6). The abrupt increase in Bowman’s capsule pressure caused by vesicoureteral reflux rapidly suppresses GFR, while bladder decompression through catheterization rapidly restores glomerular filtration. These hydrostatic events are not associated with structural changes in the kidneys. Accumulation of fluid and solutes during the brief period of RF generates strong diuretic pressure, which manifests clinically as post-obstructive diuresis. When CUR develops gradually without vesicoureteral reflux, as in phenotype two, RF most likely arises through mechanisms #3, involving progressive dilatation of the collecting system and prolonged exposure of nephrons to high hydrostatic pressure, resulting in TIF. Recovery is unlikely even after bladder decompression.

The management of patients with CUR associated with RF includes urgent and definitive treatment steps. Immediate bladder catheterization and admission to hospital are the initial treatment steps. After bladder evacuation most men (87.8% in this study) develop post-obstructive diuresis [19], through the following mechanisms:

1. Excretion of sodium and water accumulated during RF.
2. Osmotic diuresis driven by urea and other solutes accumulated during RF.
3. Reduced sodium transport in the thick ascending limb and distal tubules resulting from the prolonged obstruction.
4. Loss of the medullary osmotic gradient (medullary washout), reducing its ability to concentrate urine.
5. Reduced responsiveness of collecting ducts to ADH.

“Physiologic” post-obstructive diuresis (mechanisms 1 and 2) is not associated with dehydration or electrolyte or acid–base imbalances. On the other hand, “pathologic” diuresis (mechanisms 3–5) carries these risks [19]. In this study, electrolyte and acid–base imbalances were encountered in 7.5% of patients.

Regarding the definitive treatment, the AUA, NICE, and EUA guidelines are in consensus that patients with CUR-associated RF should not undergo a trial without catheter before prostate surgery. Surgical timing depends on the physiology of kidney, bone marrow, and bladder recovery. Renal filtration and tubular function usually begin improving within 24–72 h after bladder catheterization. Most recovery occurs within the first 2 weeks, but improvement may continue for 3–6 months; impaired urinary concentration may persist longer [20]. Bone marrow failure manifests as anemia and uremic thrombocytopathy. In the current study, anemia (defined here as hemoglobin below 11.7 g/dL—the selected cutoff distinguishing the two phenotypes) was present in 42.2% of patients. Contributing factors to anemia include hemodilution, relative erythropoietin deficiency, nutritional deficiencies of iron, folic acid, and B12, bleeding due to uremic thrombocytopathy, chronic inflammation, and shortened red-cell survival. Uremic thrombocytopathy is particularly relevant when planning prostate surgery because prostatic surgery involves significant bleeding.

As kidney function improves, hemoglobin rises gradually over weeks to months, usually reaching a plateau by three months if iron, B12, and folic acid deficiencies are corrected.

Platelet function typically recovers within 3–8 weeks after kidney function is restored.

Bladder recovery also warrants consideration. The initial bladder response to obstruction is hypertrophy and increased detrusor contractility, whereas chronic distention often leads to detrusor underactivity and decompensation [21]. Chronic overdistension damages the bladder’s connective tissue, detrusor muscle, nerves, and blood supply, progressively impairing both relaxation and contraction [22]. Following catheterization, detrusor contractility usually improves within 6–13 weeks [23]. Taken these recovery patterns together, the general recommendation for prostate surgery in most patients is around 6–8 weeks after initial diagnosis and catheterization. The findings presented in this manuscript may assist in determining a patient’s prognosis at the initial presentation.

Several small series have reported on the surgical outcomes in patients with CUR-associated RF. Hussain et al. found reported on similar maximal flow rates after TURP in 79 patients with CUR without RF and 20 with RF (16.9 ± 7.9 vs. 14.6 ± 4.1 mL/sec, *p* > 0.05), although persistence of postoperative symptoms was more common in the RF group [24]. Thomas et al. reported that, among 14 patients with CUR and RF who underwent TURP, 12 subsequently voided spontaneously, one required clean intermittent self-catheterization, and one retained an indwelling catheter [25]. Finally, Nissenkorn et al. performed prostatectomy in 26 patients with elevated serum creatinine and residual urine volumes above 1000 mL. All patients were able to void after surgery, but half had irreversible renal damage [26].

This study is limited by its retrospective, single-center design. Nevertheless, the data are relatively comprehensive, and prospective collection of a similar database would require many years. Additionally, the proposed model explaining the pathogenesis of these two clinical phenotypes, which is based on the common finding of vesico-uerteral reflux in patients with obstructive prostatic disease, remains speculative [16]. Furthermore, given the presence of two kidneys, the proposed mechanism would require simultaneous bilateral impairment of the vesicoureteral valves or the development of reflux in a single-functioning kidney after prior damage to the contralateral kidney, possibly also related to reflux.

## 5. Conclusions

Men with CUR-associated RF exhibit two clinical phenotypes: phenotype one (75% of patients) develops rapidly and resolves after bladder catheterization, whereas phenotype two (25%) develops gradually and shows minimal recovery. Despite many clinical similarities, anemia is more common in phenotype two. A hemoglobin cutoff of 11.7 g/dL distinguishes the phenotypes with 73.6% sensitivity. Phenotype one may reflect ureterovesical valve failure, with high-pressure urine reflux raising Bowman’s capsule pressure and suppressing GFR; prompt catheterization restores renal function. In contrast, phenotype two results from prolonged exposure of nephrons to elevated hydrostatic pressure and irreversible TIF. The management of men with CUR-associated RF includes immediate hospitalization and monitoring of hydration, electrolytes, and acid–base status, followed by prostate surgery 6–8 weeks later. Urologic outcomes are expected to be satisfactory, although kidney function will not recover in 25% of patients.

## Figures and Tables

**Figure 1:**
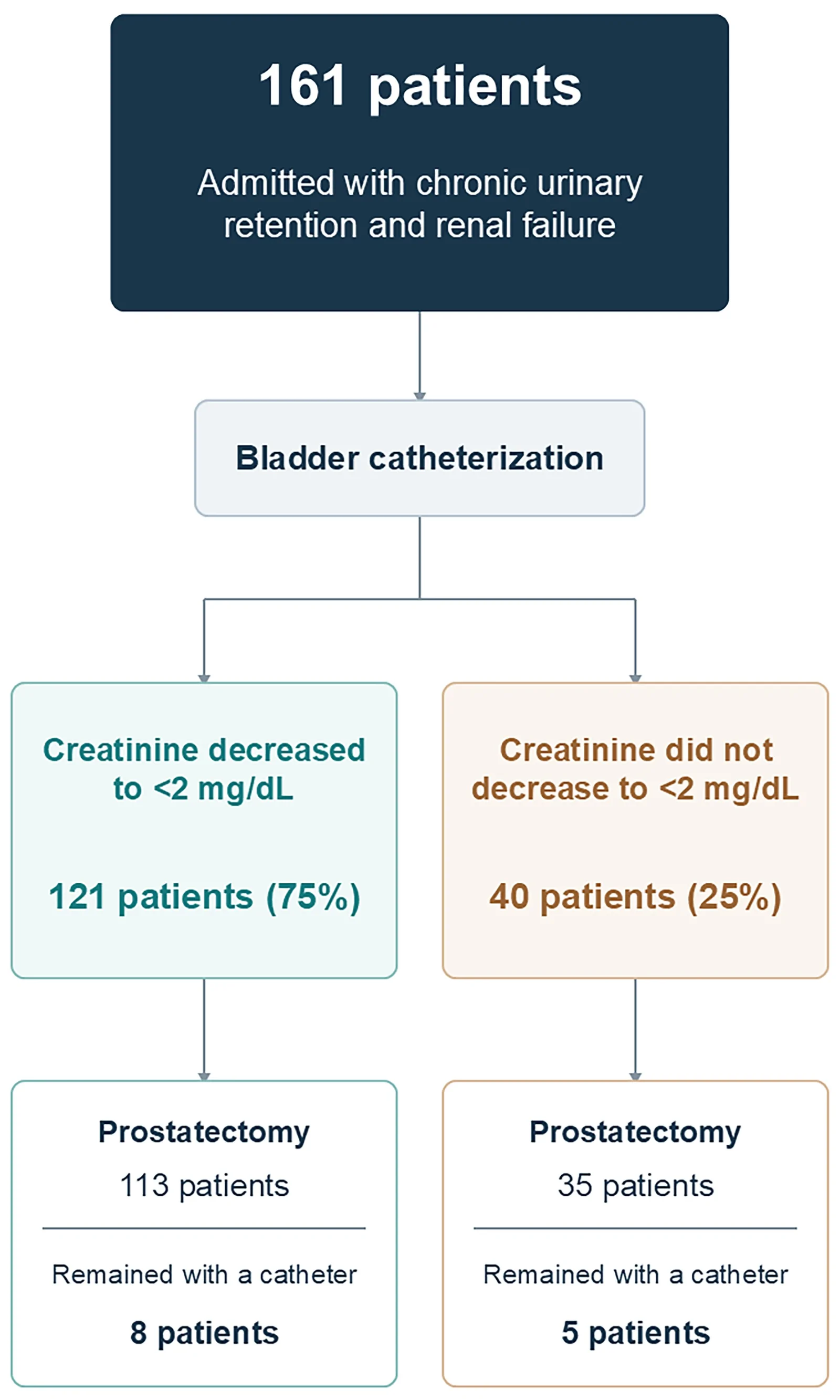
Flowchart describing the fate of patients participating in the study.

**Figure 2:**
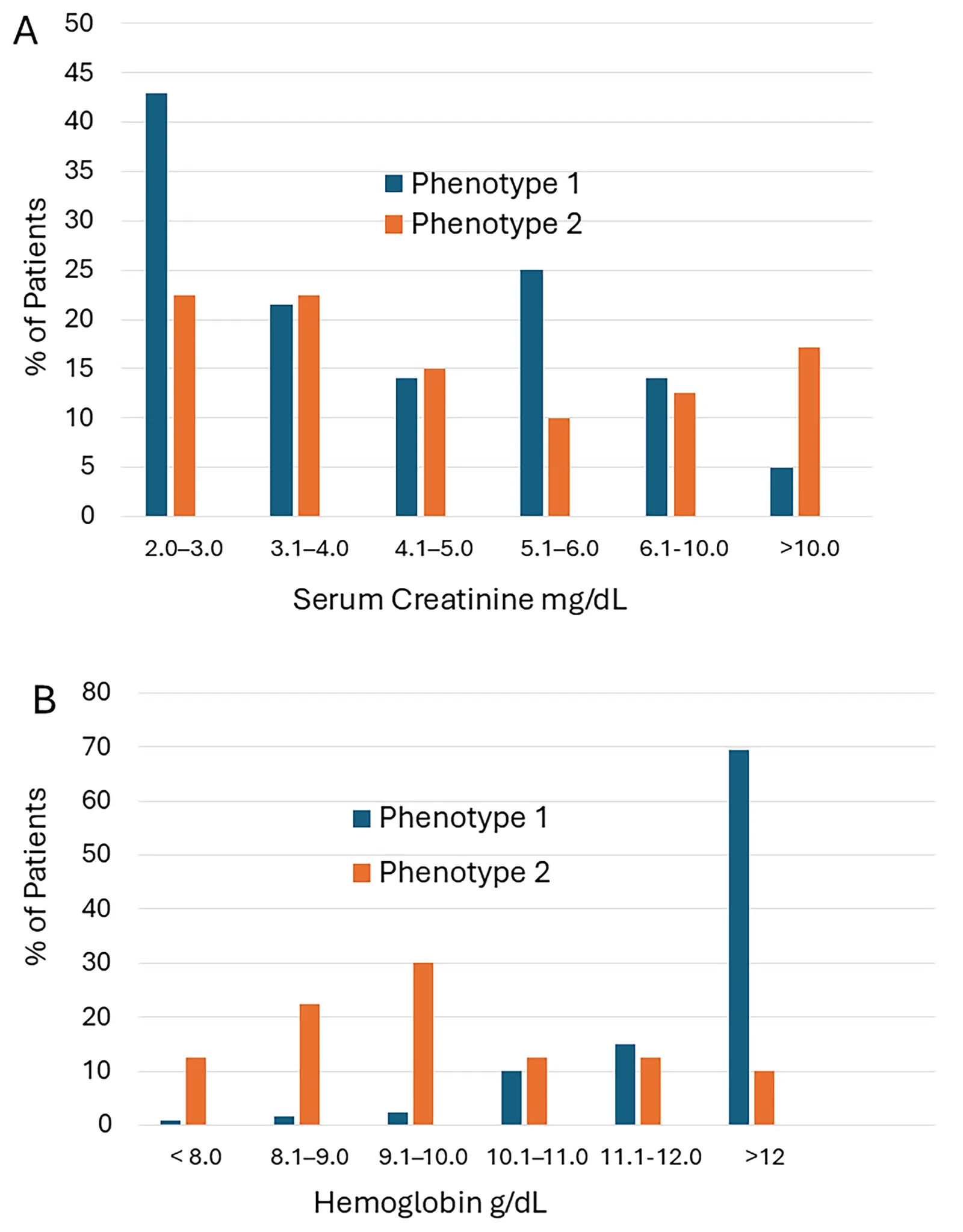
Distributions of serum creatinine (**A**) and hemoglobin (**B**) in phenotype one and two chronic urinary retention associated with renal failure.

**Figure 3:**
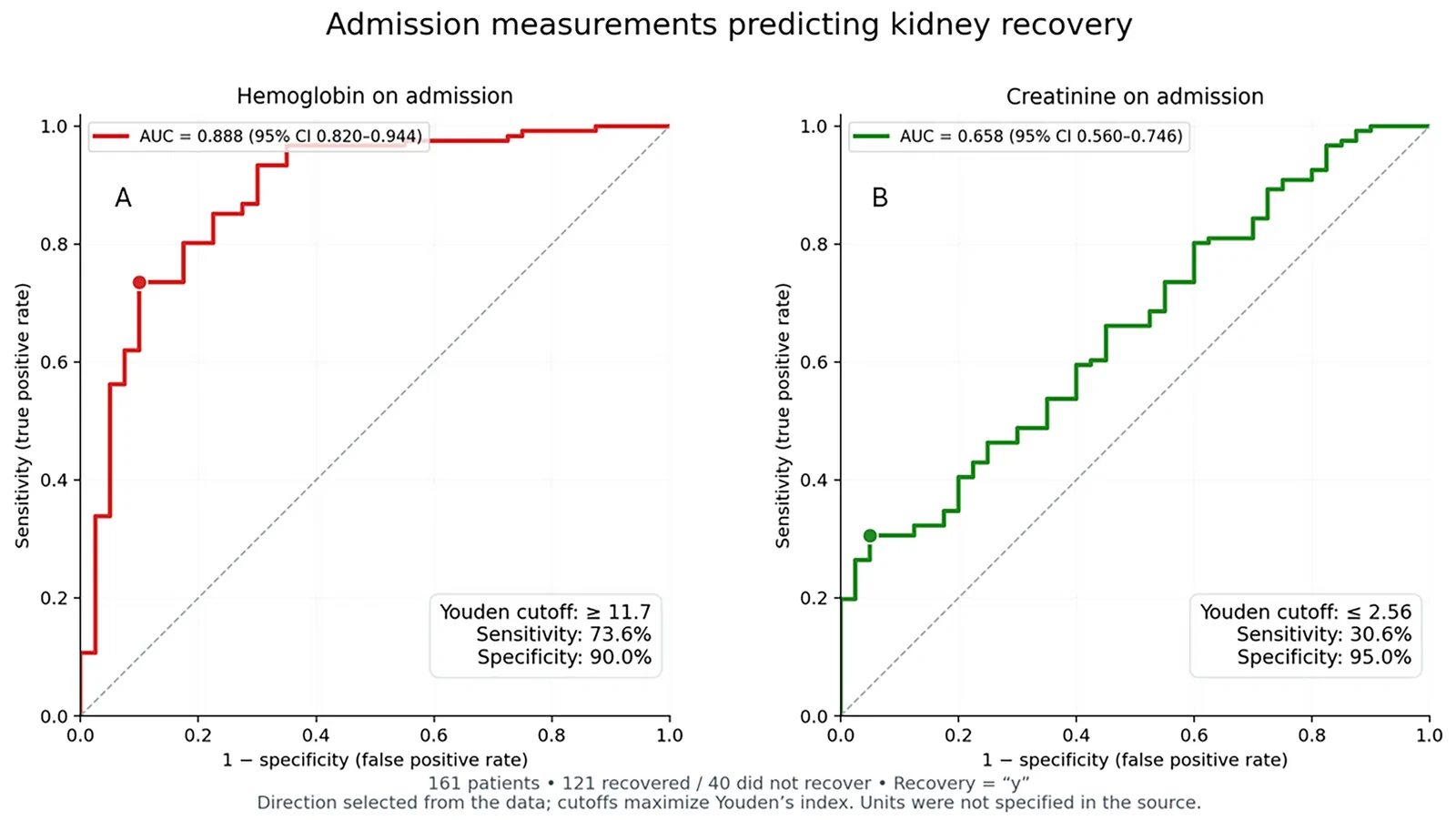
ROC curve predictions of renal recovery according to hemoglobin level (**A**) and creatinine levels (**B**) on admission. The ROC of hemoglobin provided an AUC of 0.888 (95% CI 0.820–0.944), and creatinine 0.658 (95% CI 0.560–0.746). Hemoglobin levels of 11.7 g/dL predicted phenotype one with sensitivity of 73.6%, specificity of 90%, and Youden’s J of 0.636.

**Table 1:** Characteristics of 161 patients presenting with chronic urinary retention and renal failure.

Parameter	Total
Average Age (SD)	73.6 ± 9.9
Serum creatinine upon admission (SD)	4.8 mg/dL (3.4)
GFR category at diagnosis	G3b 16 (9.9%) G4 75 (46.6%) G5 70 (43.5%)
Creatinine nadir (SD)	1.7 mg/dL (1.2)
GFR category at nadir	G1 19 (11.8%) G2 42 (26.1%) G3a 35 (21.7%) G3b 35 (21.7%) G4 22 (13.7%) G5 8 (5.0%)
Hemoglobin upon admission (SD)	12.0 g/dL (2.1)
Residual Bladder volume (SD)	1426 mL (738)
Prostate volume (SD)	88.6 mL (50.4)
Hydronephrosis	122 (75.8%)
Electrolyte/acid–base imbalances	12 (7.5%)
Post-obstructive diuresis	141 (87.8%)

**Table 2:** Characteristics of the two phenotypes of 161 men presenting with chronic urinary retention and renal failure.

	Phenotype 1	Phenotype 2	*p* Value
Number of patients	121 (75.2%)	40 (24.8%)	-
Average Age (SD)	73.9 (9.8)	72.0 (10.7)	0.34
Presence of Hydronephrosis (N/Y)	32 (25.4%)/89 (73.6%)	4 (10%)/36 (90%)	0.03
Average residual volume (SD)	1398 mL (800)	1499 mL (800)	0.48
Average prostatic volume (SD)	94 mL (44.6)	83.2 mL (44.6)	0.41
Post Obstructive diuresis (N/Y)	7 (5.8%)/114 (94.2%)	13 (32.5%)/27 (67.5%)	<0.0001
Average Serum Creatinine (SD)	4.3 mg/dL (2.6)	6.3 mg/dL (4.8)	0.01
Average eGFR (SD)	19.0 mL/min (7.9)	14.0 mL/min (7.9)	0.001
KDIGO category	G3b 16 (13/2%) G4 59 (48.8%) G5 86 (71.7%)	G4 16 (40.0%) G5 24 (60.0%)	0.115
Average Hemoglobin (SD)	12.7 g/dL (1.9)	9.8 g/dL (1.9)	<0.0001
Electrolyte imbalances following drainage (N/Y)	9 (7.4%)/112 (92.6%)	2 (5%)/38 (95%)	0.73
Average creatinine 3 months after drainage (SD)	1.3 mg/dL (1.6)	3.2 mg/dL (1.6)	<0.0001
Average eGFR 3 months after drainage (SD)	63.9 mL/min (8.2)	23.9 mL/min (8.2)	<0.0001
KDIGO category 3 months after drainage	G1 19 (15.7%) G2 42 (34.7%) G3a 35 (28.9%) G3b 25 (20.7%)	G3b 10 (25.0%) G4 22 (55%) G5 8 (20.0%)	<0.0001

## Data Availability

All raw data is available on Supplementary Materials.

## Supplementary Materials

The following supporting information can be downloaded at: https://www.mdpi.com/article/10.3390/jcm15218680/s1, Tables S1–S8: All raw data.
